# Utility of Bioluminescent Homogeneous Nucleotide Detection Assays in Measuring Activities of Nucleotide-Sugar Dependent Glycosyltransferases and Studying Their Inhibitors

**DOI:** 10.3390/molecules26206230

**Published:** 2021-10-15

**Authors:** Laurie Engel, Juliano Alves, Jacquelyn Hennek, Said A. Goueli, Hicham Zegzouti

**Affiliations:** 1 Promega Corporation, R&D Department, 2800 Woods Hollow Road, Madison, WI 53719, USA; laurie.engel@promega.com (L.E.); juliano.alves@promega.com (J.A.); jhennek@exactsciences.com (J.H.); said.goueli@promega.com (S.A.G.); 2 Exact Sciences Corporation, 5505 Endeavor Lane, Madison, WI 53719, USA; 3 Department of Pathology and Laboratory Medicine, University of Wisconsin School of Medicine and Public Health, Madison, WI 53719, USA

**Keywords:** nucleotide assays, bioluminescence, sugar substrate, fucosyltransferase, OGT, inhibitor

## Abstract

Traditional glycosyltransferase (GT) activity assays are not easily configured for rapid detection nor for high throughput screening because they rely on radioactive product isolation, the use of heterogeneous immunoassays or mass spectrometry. In a typical glycosyltransferase biochemical reaction, two products are generated, a glycosylated product and a nucleotide released from the sugar donor substrate. Therefore, an assay that detects the nucleotide could be universal to monitor the activity of diverse glycosyltransferases in vitro. Here we describe three homogeneous and bioluminescent glycosyltransferase activity assays based on UDP, GDP, CMP, and UMP detection. Each of these assays are performed in a one-step detection that relies on converting the nucleotide product to ATP, then to bioluminescence using firefly luciferase. These assays are highly sensitive, robust and resistant to chemical interference. Various applications of these assays are presented, including studies on the specificity of sugar transfer by diverse GTs and the characterization of acceptor substrate-dependent and independent nucleotide-sugar hydrolysis. Furthermore, their utility in screening for specific GT inhibitors and the study of their mode of action are described. We believe that the broad utility of these nucleotide assays will enable the investigation of a large number of GTs and may have a significant impact on diverse areas of Glycobiology research.

## 1. Introduction

Glycosyltransferases (GT) represent a large family of enzymes that belong to a well-defined enzymatic network that orchestrates the formation and maintenance of complex carbohydrate structures found abundantly in all living organisms [1]. Using activated sugars as donor substrates, glycosyltransferases transfer the sugar moiety to an array of acceptor substrates of various chemical natures, including proteins, lipids, sugars, nucleic acids, and small molecules [2]. The most common donor substrates used by glycosyltransferases are nucleotide-activated sugars, such as UDP-, GDP-, and CMP-sugars, but they can also use lipid sugar phosphates (e.g., dolichol phosphate sugar), and unsubstituted phosphates. Glycosyltransferases that use nucleotide-activated sugars are called Leloir enzymes, in honor of the 1970 chemistry Nobel Prize winner Luis F. Leloir, who discovered the first sugar nucleotide [3]. Because of the importance of the various oligosaccharide structures to cell functions, glycosyltransferases are known to be involved in a multitude of biological processes, such as cell–cell communication, immune responses [4,5,6], cell signaling and epigenetic regulation of gene expression [7,8], and plant- and bacterial-cell wall biosynthesis [9,10]. As a corollary, the disruption of these biological processes due to abnormal glycosyltransferase activity or expression can have a detrimental effect on the cell, leading to serious diseases, such as cancer, inflammation, and diabetes [11,12]. Glycosyltransferase inhibitors are being developed for the treatment of these diseases, as well as metabolic diseases, such as Morbus Gaucher, a lysosomal storage disease characterized by an accumulation of glucocerebrosides in multiple organs due to dysfunctional downstream degradation machinery (glucocerebrosidase), causing detrimental neurological and muscular symptoms [13,14]. The first-line therapy for Gaucher is Glucocerebrosidase enzyme replacement therapy, which is a burdensome treatment due to the routine injections that the patients undertake. Glucosylceramide synthase (GCS) is the GT that produces these glucocerebrosides using UDP-Glucose as a donor and ceramides as acceptor substrates. An alternative to the intense enzyme replacement therapy, the identification of a small molecule inhibitor of GCS that could reduce the glucosylceramide product in the brain and be administered orally, could be a beneficial treatment of Gaucher disease (Substrate reduction therapy) [15].

Because of the importance of this class of enzymes, there is a need to develop bioassays to study their activity and their regulation or identify chemical compounds that modulate their activity. Currently, measuring glycosyltransferase activity relies on traditional methods, such as the chromatographic separation of substrate and product or the detection of a radiolabeled product. While these assays have proved to be valuable in terms of sensitivity and precision, they are cumbersome as they require washing steps and separation of the glycosylated product for analysis and are not easily configured for rapid screening [16]. Alternatively, several assay technologies not requiring the use of radiochemicals were developed in the last two decades [17]. Some of them are fluorescence-based assays that detect the nucleoside diphosphate using either fluorescent chemosensors [18,19] or fluorescent tracers combined with immunodetection [20]. These assays have the advantage of being universal for all GTs that release the detected nucleotide. However, specificity towards the nucleotide versus the nucleotide-sugar substrate can generate higher background; thus, decreasing the sensitivity and the accuracy of the assay. Moreover, chemosensors’ availability and synthesis cost could limit their widespread acceptance [17]. Other universal nucleotide detection assays relying on the enzyme-coupled generation of fluorescence or absorbance were also developed for GT activity measurement [21,22]. The fluorescent GT assays rely on a series of coupled-enzyme reactions that use the nucleotide and generate fluorescent resorufin from the resazurin molecule using four enzymes and multiple substrates and co-factors, such as ATP, glucose, NADP+, and resazurin [21]. Due to the availability and nature of the assay components, designing these types of assays can be cost-effective. However, the number of enzymes involved, the complexity of each of the enzymatic reactions, and the multiple incubation steps required may render their implementation and their routine use challenging. Moreover, because of the increased chance that one or more of the multiple enzymes used in these assays could be prone to chemical interference from compound libraries, their use in high throughput screening could lead to high false-positive hit rates. The absorbance assay relies on a phosphatase-coupled reaction that hydrolyzes the nucleotide, and the released phosphate group is detected using a traditional colorimetric malachite green reagent [22]. Although assays relying on absorbance readout can be adapted to 96-well plate formats, they are not sensitive enough as they require high reaction volumes and high inorganic phosphate to be generated to create a signal above the background. Another reason for its low sensitivity is the high background generated due to the presence of inorganic phosphate contamination in many common buffers and reagents used in the enzyme reactions. Thus, their low sensitivity precludes them from detecting low activity enzymes and makes them not easily adaptable to high-density plate formats that require low reaction volumes [23]. Other technologies that employ fluorescently labeled donor or acceptor substrates were also developed for glycosyltransferase activity, or inhibitor binding determination. These assays can rely on FRET technology, where fluorescence energy is transferred from a fluorescent donor to a fluorescence acceptor emitting a signal in a defined wavelength after the fluorescent sugar is transferred by the GT [24,25]. Another method uses fluorescent ligand displacement where a low fluorescence sugar donor probe is bound to the GT, and upon binding of a competitive small molecule compound to the donor pocket, a change in fluorescence or fluorescence polarization occurs [26,27]. While these technologies are simple and well suited for HTS, they are not applicable to all glycosyltransferases because of the need to synthesize and optimize specific fluorescent donors and/or acceptors for each GT to be studied, or they are only used to determine compound binding and not for GT activity assessment [26]. Moreover, there is no robust assay that can be easily used to characterize the family of phosphoglycosyltransferases due to their nature of being localized in the membrane, the difficulties associated with their expression and purification, and the challenge of synthesizing labeled versions of their substrate to use in activity analysis [28].

Although these assays have been used successfully to characterize glycosyltransferase activities, most still suffer from a variety of limitations that make them difficult to address all the needs of GT activity determination without relying on lengthy protocols, use of hazardous radiochemicals, special reagent synthesis, or the requirement of specialized detection instruments. Here we describe the use of a suite of bioluminescent nucleotide detection assays for measuring GT activities based on UDP, GDP, UMP, and CMP detection. Each of these assays is performed in a one-step detection that relies on simultaneously converting the nucleotide product of any GT to ATP and the latter into light in a luciferase reaction. In a Leloir-type glycosyltransferase reaction, using a nucleotide-sugar donor, the enzyme transfers the sugar to an acceptor substrate and the nucleotide moiety is released as a product. Therefore, an assay that detects the nucleotide molecule could be universally used to assess the activities of all these glycosyltransferases in vitro. In fact, many enzymes other than GTs also utilize nucleotides as substrates or generate them as reaction products. These enzymes are widely studied, and some are validated drug targets. Thus, assays that monitor the activity of these enzymes are desirable in the search for selective modulators and the development of novel therapeutics. Each nucleotide is a common product of a large group of enzymatic reactions, such as glycosylations. The development of detection assays that monitor nucleotide production with high performance and in a homogeneous format will expand the number of enzymes that could be investigated and will have a significant impact on diverse areas of research. The bioluminescent-based assay platform we developed is robust and can monitor the concentrations of various nucleotides as a readout for the corresponding enzyme activity. The nucleotides are converted into a robust enzymatic reaction to ATP and then detected using a Luciferase/luciferin reaction to generate bioluminescence. A few examples include bioluminescent ATP and ADP detection assays that were validated in monitoring the activity of many drug targets, including kinases, ATPases, and helicases [29,30,31,32]. An AMP detection assay was used to measure AMP as a product of diverse biochemical reactions, such as ubiquitin ligases, DNA ligases, and cAMP-dependent phosphodiesterases [33,34]. GTPases and their regulators have been challenging to study due to the scarceness of convenient and easy-to-use assays. Using this core technology, a bioluminescent GTP detection assay was developed to monitor the activities of these important drug targets and their immediate regulators [35,36]. This core bioluminescent technology employs a luciferase variant called Ultra-Glo that, in combination with the reagent formulation, proved to be simple, sensitive, and resistant to chemical interference during HTS for pharmacologically active compounds identification [37].

Here we demonstrate the application of this same platform to develop luciferase-based nucleotide assays for glycosyltransferase activity detection, and we demonstrate their utility in studying the specificity of transfer of different sugars to different acceptors by glycosyltransferases from different families. These bioluminescent assays were shown to be adequate for determining enzyme kinetic parameters, such as Km for donor and acceptor substrates, and for identifying GT small molecule modulators. We demonstrate that this generic GT assay platform can be used to characterize GTs from different families, such as GlcNAc transferases, fucosyltransferases, sialyltransferases, and the hard to analyze phosphoglycosyltransferases.

## 2. Results and Discussion

### 2.1. Bioluminescent Glycosyltransferase Assay Principle and Formats

**Figure 1:**
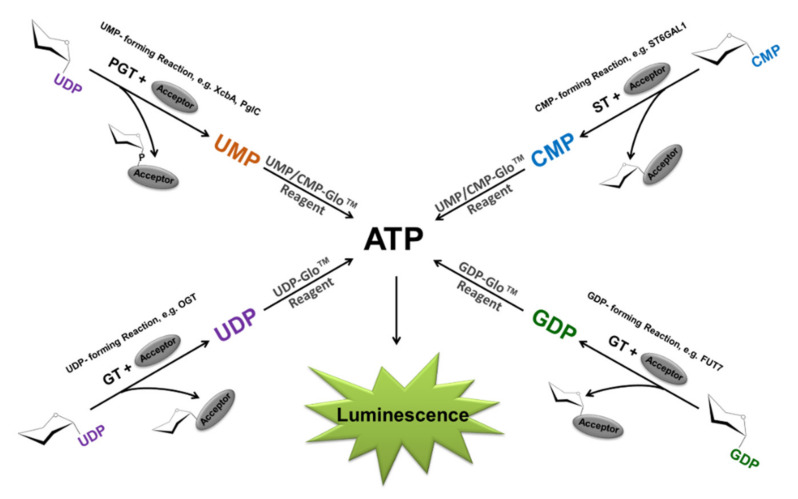
Bioluminescent nucleotide assays principle. UDP, GDP, UMP/CMP-Glo assays detect the corresponding nucleotides generated as a result of glycosyltransferase activity. The Glycosyltransferase Glo assays are performed in one step after the completion of the GT reaction. The nucleotide-Glo reagents contain a converting enzyme specific for either UDP, GDP, or UMP/CMP that converts the produced nucleotide to ATP. Simultaneously, the newly formed ATP is used by the luciferin/luciferase system to generate luminescence. The light generated correlates to the nucleotide present and glycosyltransferase activity.

A bioluminescence-generated chemical/biochemical reaction requires three components, the luciferase enzyme (e.g., Firefly luciferase), luciferin, and ATP. The enzyme catalyzes luciferin oxidation using ATP and molecular oxygen to yield oxyluciferin, which emits light upon a change in its energy state [38]. In general, the light generated by firefly luciferase is proportional to the concentration of these three components. Bioluminescent assay development over the years was based on measuring one of the components of this reaction as a means of detecting cellular or biochemical events while keeping the other two reaction components constant. Depending on the biological event to be investigated, the assay can be configured to detect variable amounts of the enzyme (luciferase genetic reporters), luciferin (non-light-emitting pro-luciferin substrates that get converted to luciferin through the action of specific enzymes of interest) [39], and finally, ATP itself as the other substrate of luciferase. ATP-based bioluminescent assays have been widely used to detect cell viability or to detect the biochemical activity of enzymes that either uses ATP as a substrate or produce it as a product. The bioluminescent glycosyltransferase assays (Glo assays) used in this study take advantage of the latter. A Leloir GT uses an activated nucleotide-sugar as a substrate donor for glycosylation of a substrate acceptor and releases the nucleotide as a secondary product. As shown in Figure 1, all the Glycosyltransferase-Glo assays are performed in one step after the completion of the GT reaction. An equal volume of the specific nucleotide-Glo reagent, which contains a converting enzyme specific for either UDP, GDP, or UMP/CMP, is added to the GT reaction to convert the produced nucleotide to ATP. Simultaneously, the newly formed ATP is used by the luciferin/luciferase components of the reagent to produce bioluminescence (Figure 1). The amount of light generated is proportional to the nucleotide produced and to the activity of the glycosyltransferase. The incubation time of the reagent was optimized to 60 min to allow full conversion of the nucleotide to light and generate a linear relationship between the number of nucleotides present and light output.

### 2.2. Glycosyltransferase Assays Sensitivity and Linearity

**Figure 2:**
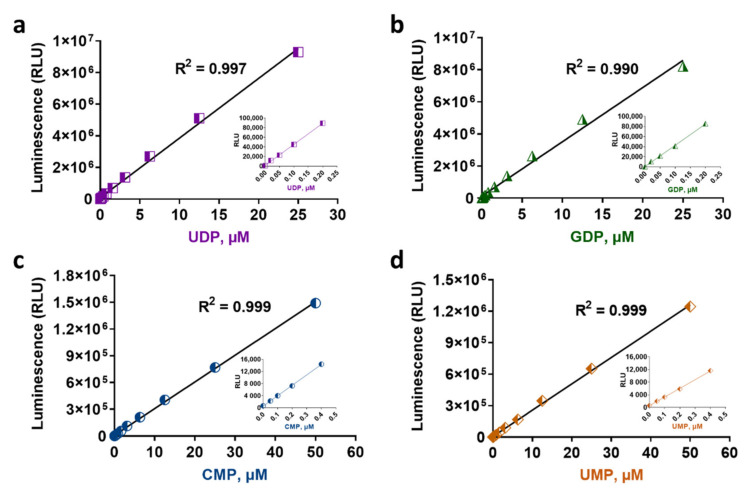
Linearity and sensitivity of bioluminescent nucleotide assays. The UDP-Glo (**a**) and GDP-Glo (**b**) assays can detect up to 25 µM, and the UMP/CMP-Glo (**c**,**d**) can detect up to 50 µM of the corresponding nucleotides. Luminescence values represent the mean of three replicates. RLU = relative light units.

All GT-Glo assays require a 60-min incubation to reach the maximum light output. In this time frame, the UDP- and GDP-Glo assays can detect up to 25 µM, and the UMP/CMP-Glo can detect up to 50 µM of the corresponding nucleotide (Figure 2). This detection range meets the requirement of a wide range of GT enzyme activities (data not shown). All the assays are simple to perform following the addition pattern of a 1:1 ratio of the GT reaction: Nucleotide-Glo Reagent, with example volumes 25:25 µL used for 96-well plates shown here and volumes of 10:10 µL or 5:5 µL used for 384-well plates (data not shown).

To assess the linearity and sensitivity of the bioluminescent nucleotide detection, we performed a serial dilution of the nucleotides UDP, GDP, UMP, and CMP in 96-well plates to create a standard curve and detected the light generated by each concentration following the assay procedure described in the Materials and Methods section. Figure 2 and Table 1 show the standard curves generated, the luminescence values in relative light units (RLU), and the signal to background ratios (S/B) resulting from each nucleotide concentration detection. There is a linear response with increasing concentrations of each nucleotide using the corresponding detection reagent. The nucleotide-Glo assays can detect the corresponding nucleotide in the linear range up to 25 or 50 µM (Figure 2) with an R^2^ value of 0.99. These assays are also sensitive with a limit of detection of approximately 1–5 nM for UDP and GDP or 50 nM for UMP or CMP detection (Table 1). The stability of the signal was assessed by recording the luminescence emitted from the same standard curve every hour after the first read, and it was found that the RLU signals remain stable for at least 3 h at room temperature (data not shown). It should be noted that the detection of other nucleotides was also tested, and it was found that similar to the UMP/CMP-Glo that can detect both UMP and CMP, the UDP-Glo can detect UDP and CDP with the same performance (Table 1) and may be used to detect the activity of enzymes that release CDP as a product (data not shown).

The range of detection and the sensitivity of the assays shown here would meet the requirements of activity detection for a broad range of GT enzymes and because of their homogeneous nature (add and read with no washes and no liquid transfers), and the stability of the signal generated, these bioluminescent GT assays are ideal for high throughput screening where the batch processing of plates may be required.

### 2.3. Characterization of Diverse Glycosyltransferase Activities

**Figure 3:**
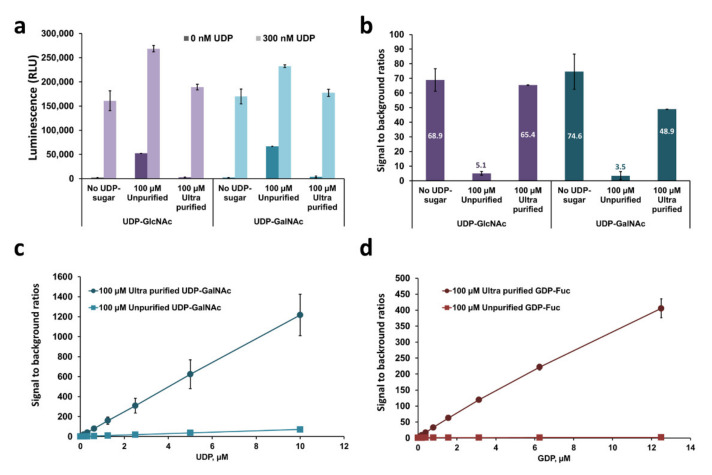
Effect of the sugar substrates purity on the Glo assays performance. Luminescent signal (**a**) and sensitivity (**b**) of the UDP-Glo in the absence or presence of unpurified and ultra-pure sugar substrates. (**c**,**d**) Standard curves of UDP and GDP detected with of UDP-Glo and GDP-Glo, respectively, in the presence of unpurified or ultra-purified sugar substrates.

Most glycosyltransferases in all organisms use activated sugars that are conjugated to mono or diphosphate nucleotides as sugar donor substrates. After the sugar transfers to an acceptor substrate, the nucleotide moiety is released. Because the GT-Glo assays detect nucleotide generation as a universal product, they would be able to measure the activity of diverse GTs that produce these nucleotides as a product. We wanted to test the performance of these assays in detecting various GT activities. We found that commercially available substrates are contaminated with free nucleotides due to their instability and autohydrolysis, which would increase the background luminescence in the assay. Therefore, ultrapure and stable sugar-nucleotide donors are required to minimize luminescence background levels and increase the sensitivity of the assays. The ultrapure sugar substrates available with the assays are known to have very minor nucleotide contamination due to the manufacturer’s in-process purification, buffer, and storage conditions (less than 0.007% for UDP-sugars and less than 0.035% for GDP-sugars). The assays were shown to be sensitive when testing nucleotides in a pure system (Figure 2). To assess the effect of the sugar substrates purity on the Glo assays performance, we compared the signal and sensitivity (signal over background ratios) of the UDP-Glo and GDP-Glo in detecting the corresponding nucleotides in the presence of unpurified and ultra-pure sugar substrates. UDP detection was used to detect 300 nM UDP in the absence or presence of unpurified or ultra-pure 100 µM UDP-GlcNAc or UDP-GalNAc. As a control, the background was assessed in the absence of added UDP (0 nM UDP). When no sugar substrate was present, there was a relatively low assay background signal at 0 nM UDP and a signal over 150,000 RLU generated from 300 nM UDP (Figure 3a). This produced a signal-over-background ratio (SB) close to 70-fold (Figure 3b). When unpurified sugar was added at 100 µM, both the background and the signal increased dramatically, resulting in a significant decrease in the SB ratio to ~5 fold, which lowered the assay sensitivity. Both UDP-GlcNAc or UDP-GalNAc generated similar results. On the contrary, when ultrapure sugar preparations were added at the same concentration of 100 µM to the 0 and 300 nM UDP samples, they had no noticeable effect on either the background or the signal RLUs. The RLUs resemble those of the samples with no sugar substrate added, resulting in a recovery of the high SB ratios and the assay sensitivity (Figure 3a,b). Moreover, we also compared the effect of both unpurified and ultrapure UDP-GalNAc and GDP-Fucose on the sensitivity of UDP-Glo and GDP-Glo assays, respectively, using an eight-point standard curve. Similarly, when non-purified sugars were added, there was a great decrease in sensitivity, as evidenced by very low SBs (Figure 3c,d).

To obtain meaningful results when using nucleotide detection assays (Glo or other), it is important to use purified sugars, not only to ensure a great assay sensitivity and dynamic range but also to study GT activities under optimal reaction conditions void of any nucleotide product at the start of the enzyme reaction that is known to have an inhibitory effect on GTs (product feedback inhibition). Although there are only a few ultrapure nucleotide sugars commercially available as a substrate (UDP-Glc, -Gal, -GlcNAc, -GalNAc, -GA, GDP-Fuc, and GDP-Man), any nucleotide sugar can be cleaned with a simple method prior to its use in the Glo assays. For example, Calf Intestinal Alkaline Phosphatase (CIAP) was used to degrade the free nucleotides, followed by removal of the enzyme using a microcentrifuge concentrator enzyme [40].

**Figure 4:**
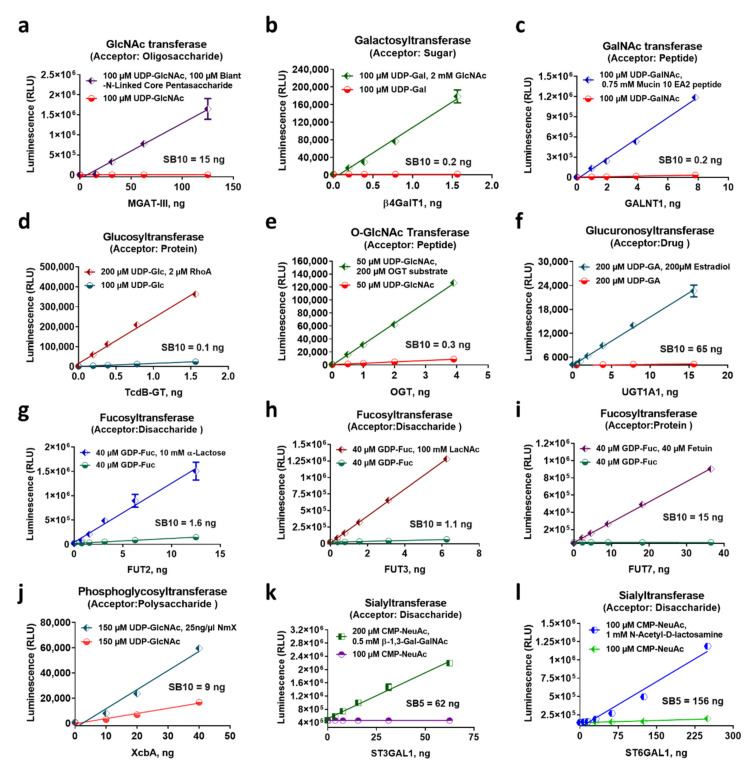
Universality of bioluminescent nucleotide assays towards glycosyltransferases. Enzyme titrations of representative members of each of the nucleotide forming GT subfamilies in the presence of the activated sugars and in the presence or absence of the indicated acceptor substrates of different chemical structures. (**a**–**f**) UDP detection in glycosyltransferases using UDP sugars. (**g**–**i**) GDP detection of fucosyltransferase activities using GDP-fucose. (**j**) Detection of UMP in the phosphoglycosyltransferase XcbA reaction using UMP/CMP-Glo. (**k**–**l**) Detection of sialyltransferase activity with UMP/CMP-Glo. Reactions were performed in duplicates. Results shown are means ± standard deviations. Names of the GTs used are indicated on the *x*-axis.

To evaluate assay performance in monitoring the biochemical activity of diverse GT enzymes and ensure their universality, we tested several members of the nucleotide-sugar-dependent Glycosyltransferase superfamily. Representative members of UDP-sugar utilizing GTs, such as MGAT-III, β-4GALT1, UGT, and OGT, or the phosphoglycosyltransferase XcbA, as well as representatives of GDP-sugar utilizing enzymes (fucosyltransferases FUT2, 3 and 7), and sialyltransferases, such as ST3GAL1 and ST6GAL1, were tested using with their respective nucleotide-sugar donor and acceptor substrates highlighted in Figure 4, and nucleotide generation was detected using the corresponding Glo assay. In the presence of the corresponding substrates, the enzymes generated varying amounts of their specific nucleotide in a concentration-dependent manner. Thus, an increase in each of the nucleotide production was proportional to the increase in the amount of the GT enzyme used (Figure 4). The nucleotides were detected with high sensitivity as indicated by the range of signal to background ratios (SB) generated (shown by their SB5 or 10 values), confirming that nucleotide detection is adequate for monitoring enzymes with varying specific activities. The most active enzyme tested was the glucosyltransferase TcdB-GT that generated an SB of 10 with only 0.1 ng of the enzyme (Figure 4d), and the enzyme with the lowest activity detected was the sialyltransferase ST6GAL1, which generated an SB of five with 156 ng of enzyme (Figure 4l). It should also be noted that the assays were able to detect the GT activities with any type of acceptor substrate (i.e., peptide, protein sugar, or drug), confirming the universality of the nucleotide-Glo assays (Figure 4). Unlike other methods that detect the glycosylated product and require a modified substrate for each enzyme to allow an output signal detection, such as fluorescence after sugar transfer, we demonstrated here the usefulness of a generic bioluminescent-based nucleotide detection method for the in vitro characterization of virtually any glycosyltransferase.

### 2.4. Profiling GT Substrate Selectivity with Nucleotide Detection

**Figure 5:**
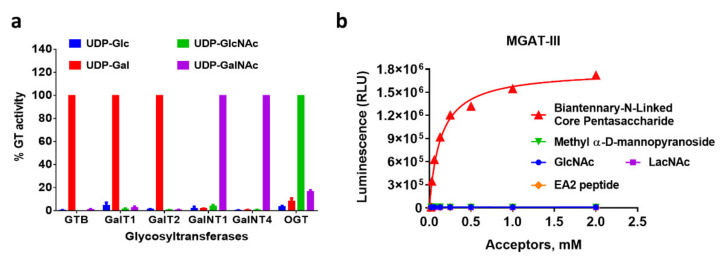
Determination of glycosyltransferases preference for specific nucleotide-sugar donor and acceptor substrates. (**a**) UDP-Glo detection of UDP-sugar specificity for six glycosyltransferases at one single substrate concentration. (**b**) UDP-Glo detection of acceptor substrate specificity for MGATIII using a titration of multiple substrates of different structures and the sugar donor UDP-GlcNAc.

Because these assays can detect the activity of any nucleotide-sugar-dependent glycosyltransferase that produces the corresponding nucleotide, regardless of the acceptor substrate chemical structure, they could potentially provide a powerful strategy for specifying the nature of donor and acceptor substrates used by putative GT enzymes or validate the acceptor selectivity of known GTs. Using UDP-Glo assay as a model for this application, we tested six GT enzymes that are known to use one specific UDP-sugar to confirm that the bioluminescence is generated only when that specific UDP-sugar is used as a substrate. Each of the GTs were incubated with their acceptor substrate, and each of the donor sugar substrates, UDP-Glc, UDP-GlcNAc, UDP-Gal, and UDP-GalNAc, were used in four separate reactions for each enzyme. Figure 5a shows that only when the specific sugar donor substrate is present in the GT reactions performed luminescence was produced. GTB, which is a glucosyltransferase, generated luminescence with UDP-Glc and both galactosyltransferases GalT 1 and 2 used UDP-Gal exclusively to generate UDP (Figure 5a,b) and the N-acetylgalactosaminyltransferases GalNT 1 and 4 were selective for UDP-GalNAc. OGT, which is an O-GlcNAc transferase, generated the maximum light output using UDP-GlcNAc consistent with its function. However, OGT could also use UDP-GalNAc as a substrate with less than 20% activity compared to UDP-GlcNAc, similar to what was previously reported using a radiocapture assay [41]. We also show that OGT could use UDP-Gal as a substrate but only with ~10% activity compared to UDP-GlcNAc (Figure 5a). We then tested the UDP-Glo assay to analyze the acceptor substrate specificity by using β-1,4-mannosyl-glycoprotein 4-β-N-acetylglucosaminyltransferase MGAT-III as an example. This GT enzyme catalyzes the addition of a single GlcNAc to the β-linked mannose of the trimannosyl core of N-linked sugar chains producing a bisecting N-acetylglucosamine (GlcNAc). MGAT-III was incubated with its specific sugar donor UDP-GlcNAc in the presence of a titration of different known sugar acceptor substrates with different chemical structures, including two monosaccharides, a disaccharide, and a peptide. In one of the reactions, a biantennary N-linked core pentasaccharide was used as the sugar acceptor (Figure 5b). After the reaction, UDP production was detected with a UDP-Glo assay. As predicted, MGAT-III could use only the substrate containing the beta-linked mannose to transfer the GlcNAc and produce luminescence in a substrate-dependent Michaelis–Menten-type curve (Figure 5a).

While we used known glycosyltransferases to demonstrate donor/acceptor substrate preferences, others have shown the importance of these assays in unlocking the glycosylation specificity of GTs of unknown mechanisms [42,43,44,45], characterizing the biochemical features of difficult-to-assay PGTs and their homologs from different species [46], or screen various naturally-occurring substrates of plant UGTs [47]. Using UDP-Glo assay, TMEM5, which is a membrane protein required for the functional glycosylation of dystroglycan, was shown to be a xylosyltransferase by testing multiple UDP-sugars, and only the UDP-Xylose was used by the protein [44]. Sugar acceptor selectivity of protein O-linked mannose β-1,4-N-acetylglucosaminyltransferases POMGNT1 and POMGNT2, was also investigated using synthetic α-dystroglycan glycopeptides and validated with UDP-Glo assay [42]. The kinetic parameters of these enzymes and the tested peptides were determined using the bioluminescent UDP detection assay. In this study, the authors measured Km, kcat, and kcat/Km for the synthetic glycopeptides with POMGNT2 and found that the data is consistent with the results obtained in other assays used, such as radioactivity and mass spectrometry transfer assays [42]. Finally, using UMP-Glo assay, determination of the activity of three different bacterial PGTs (PglCs from H. pullorum and C. jejuni and WecA from T. maritima) was carried out, and their kinetic parameters were compared and found to be consistent with radioactivity-based assay without the requirement of preparing specialized radiolabeled UDP-sugars [46].

### 2.5. Determination of Enzyme Kinetic Parameters

**Figure 6:**
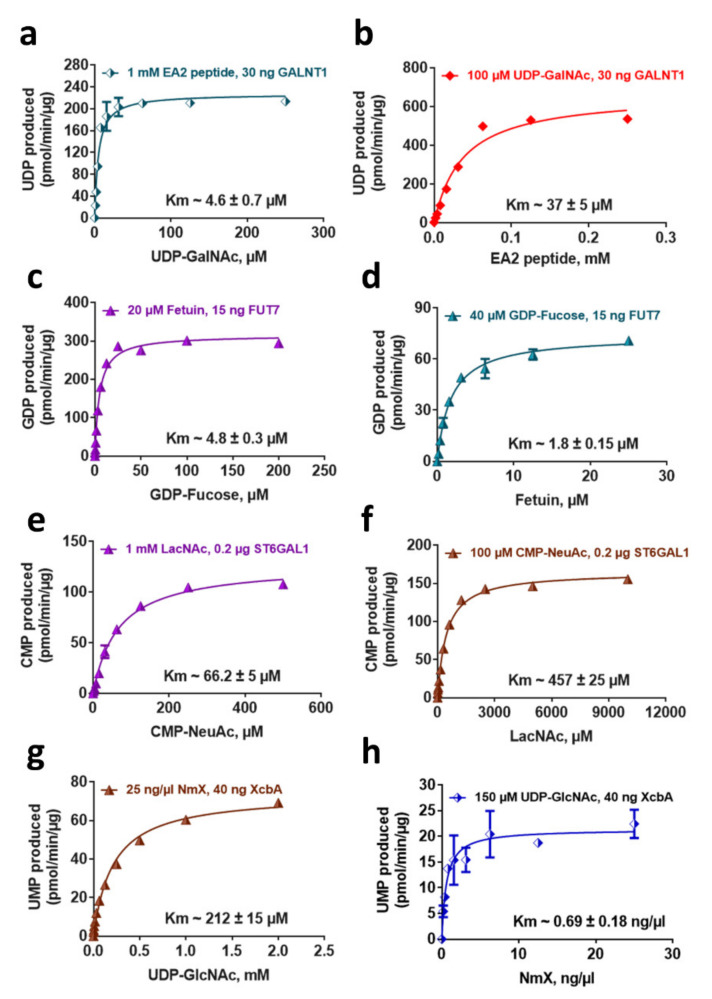
Substrate kinetic analysis of glycosyltransferase reactions using bioluminescent nucleotide assays. (**a**,**c**,**e**,**g**) Km determination of the four nucleotide sugars in GalNAc, Fucosyl, Sialyl, and phosphoGlcNAc—transferase reactions using the indicated concentrations of the corresponding acceptor substrates. (**b**,**d**,**f**,**h**) Km determination of the different acceptor substrates in GalNAc, Fucosyl, Sialyl, and phosphoGlcNAc—transferase reactions using the indicated concentrations of the corresponding sugar donor substrates. The reactions were performed in duplicates, and the results shown are means ± standard deviations. Km values were extracted from the data after fitting to the Michaelis–Menten equation using the non-linear regression fit in GraphPad Prism^®^, version 9.

The bioluminescent assays detect nucleotides in a linear fashion up to 25 µM for UDP and GDP and 50 µM for CMP and UMP. To perform a biochemical GT enzyme reaction, it is important to know the requirement for both substrates’ concentrations. Because these bioluminescent assays detect the product released from the sugar donor, there are no limitations on the acceptor substrate concentrations used in the assay. However, it is crucial to use a concentration of the nucleotide-sugar donor substrate that is both unlimiting to the enzymatic reaction rate of substrate conversion and still allows the generation of a nucleotide concentration that can be detected in the linear range of the assay. Based on the glycosyltransferases tested, this detection range is sufficient for measuring virtually any GT activity level (Figure 4). To assess the extent of substrate concentrations that can be used, we tested GT enzymes representing the four nucleotides that can be detected by these assays and titrated one of their acceptor or donor substrate in the presence of an unlimiting amount of the other substrate and calculated the apparent Km values of these substrates (Figure 6). The results show that the Km values vary depending on the enzymes and substrates used, and more importantly, that the concentration of nucleotides produced is within the assays’ detection range. This suggests that even in the presence of high nucleotide sugar concentration, the maximal conversion to nucleotide can easily be detected with the bioluminescent assays described here. These results also show that these assays are useful for determining enzyme kinetic parameters where multiple variables can be assessed easily in one experiment. It is noteworthy that it is important to determine these parameters when comparing different substrate’s requirements for a GT enzyme and when selecting substrate concentration for an inhibitor compound screening. It is common to use two to four times the donor Km to ensure comparability of different compound potencies and when different enzymes are profiled [48]. The potency of substrate-competitive inhibitors is affected by the affinity of the enzyme for the donor substrate and its concentration. Hence the need for using the right substrate concentration in the reaction.

### 2.6. Characterization of GT Acceptor-Dependent and -Independent Nucleotide-Sugar Hydrolysis

Transferases are enzymes that generally use metabolic donors, such as ATP, acetyl-CoA, and nucleotide-sugars, to transfer the small molecular groups, e.g., phosphoryl, acetyl, and glycosyl, to an acceptor substrate of any chemical structure, e.g., protein, peptide, or sugar. Because transferases, such as GTs, have two substrates, they generate two products, and assays can be used to detect either product. Assays that detect the modified acceptor substrate, such as radioactive and mass spectrometry assays, report only on the transferase activity of the enzyme and do not show the level of the donor substrate conversion, which could represent a mix between acceptor-dependent and -independent donor substrate hydrolysis. With the type of assays that detect the secondary product of the transferase reaction, such as the nucleotide-based bioluminescent assays, it is possible to assess the level of acceptor-independent donor substrate hydrolysis. In earlier studies, we and others reported on the fact that many of the transferases, including kinases, hydroxylases, and glycosyltransferases, could hydrolyze the donor substrate in the absence of the acceptor substrate [49,50,51]. In fact, this could be an advantage during assay development for transferases that display measurable intrinsic hydrolase activity, as there is no need for an acceptor substrate to be added to the enzymatic reaction components. Furthermore, this hydrolase activity was used successfully in high throughput screening for compound inhibitors for kinases and for assessing the type of sugar donor molecules for putative glycosyltransferases [40,52,53]. UDP-Glo was shown specifically in this application, where the GT hydrolase activity was monitored to assess the optimal reaction conditions of a GT without the knowledge of its acceptor substrate [40]. Here we show that nucleotide formation was also detected for many GT enzymes tested in the absence of an acceptor substrate, especially when higher enzyme amounts are used in the reaction (Figure 4). Nevertheless, significantly higher enzymatic activity in the presence of the acceptor substrate was detected. We believe that this enzyme hydrolase activity only happens in vitro as in the absence of an acceptor, the enzyme catalyzes a transfer of the sugar moiety to a water molecule releasing the nucleotide.

**Figure 7:**
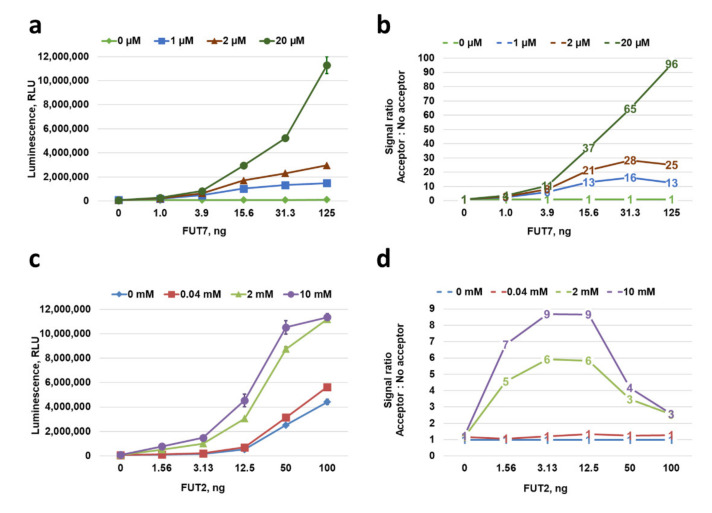
Detection of acceptor substrate-dependent and -independent GDP-Fucose hydrolysis of FUT7 and FUT2 enzymes. (**a**,**c**) Luminescence signal generated from GDP-Fucose hydrolysis by FUT7 and FUT2 enzyme titrations in the absence or presence of different concentrations of the acceptor substrate Fetuin or LacNAc, respectively. (**b**,**d**) Signal windows generated with each enzyme and acceptor substrate concentrations showing the absence (FUT7) or presence (FUT2) of intrinsic acceptor-independent GDP-sugar hydrolase activity.

To investigate this event further and establish reaction conditions to differentiate between acceptor-dependent and -independent nucleotide-sugar hydrolysis for GTs that have intrinsic hydrolase activity, we selected two fucosyltransferases FUT2 and FUT7 that showed some or no noticeable hydrolase activity in the absence of acceptor substrate, respectively (Figure 4). Both FUTs were tested in the absence or presence of increasing concentrations of their corresponding acceptor substrates to determine at what substrate and enzyme concentrations an activity window can be assigned to a substrate-dependent activity (Figure 7). FUT7 did not produce GDP at any enzyme concentration tested in the absence of its acceptor Fetuin, and it shows an increase in activity with increasing concentrations of the acceptor up to 20 µM (Figure 7a). This is consistent with the Michaelis–Menten curve of FUT7 in Figure 6 that showed a Vmax activity was reached with any concentration of Fetuin above 10 µM. It also produced increasing windows of activity represented by the acceptor:no-acceptor signal ratio (Figure 7b), indicating that to set up a FUT7 biochemical reaction using GDP-Glo assay, any concentration of Fetuin above 10 µM can be used to detect acceptor-dependent FUT7 transferase activity. On the other hand, FUT2 hydrolyzed GDP-Fucose and produced a background GDP in the absence and also in the presence of 40 µM of its acceptor α-Lactose, indicating that at this acceptor concentration we cannot differentiate between the hydrolase and the acceptor-dependent transferase activities of FUT2 shown by the lack of activity window represented by the acceptor:no-acceptor signal ratio (Figure 7c,d). However, by increasing the α-Lactose concentration above 2 mM, FUT2 had an activity close to Vmax (data not shown) and the activity window represented by the acceptor:no-acceptor signal ratio increased significantly, allowing detection of an acceptor-dependent FUT7 activity. It should be noted that although the activity of FUT2 increased in the presence of the acceptor substrate, we cannot exclude that some of the GDP detected could still be a product of GDP-Fucose hydrolysis with no associated transfer. Nevertheless, to set up an optimal FUT2 biochemical reaction using a GDP-Glo assay, a concentration of α-Lactose above 2 mM should be used to ensure the detection of acceptor-dependent FUT2 transferase activity. In addition, this experiment also showed that at a lower amount of the enzyme, the GDP-Fucose hydrolysis is less prominent, resulting in a higher acceptor:no-acceptor signal ratio (Figure 7c,d). Therefore, in addition to a higher acceptor substrate concentration, it is preferable to also use a lower amount of enzyme in order to detect more acceptor-dependent FUT2 transferase activity.

### 2.7. Glycosyltransferase Inhibition Assays

Because of their homogeneous nature, bioluminescent biochemical assays can be adapted very easily to high throughput screening for compound inhibitors. To demonstrate the bioluminescent nucleotide assays described here as a useful strategy for glycosyltransferase inhibitor identification, we tested the inhibition of two GTs with different nucleotide-activated sugars using UDP-Glo and GDP-Glo bioluminescent assays.

**Figure 8:**
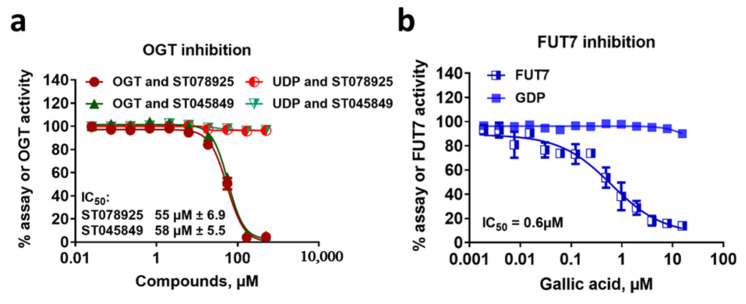
Detection of glycosyltransferase inhibitor effect using bioluminescent nucleotide assays. (**a**) Inhibition of OGT by two compounds detected with UDP-Glo assay. (**b**) Inhibition of FUT7 Gallic acid detected with GDP-Glo assay. To control for assay reagent inhibition with the compounds, a titration of the compounds was performed in the presence of the nucleotide with no enzyme. Curve fitting and IC_50_ value determinations were performed using GraphPad Prism^®^ version 9, sigmoidal dose-response (variable slope) software. Reactions were performed in duplicates, and the results shown are means ± standard deviations.

We evaluated OGT and FUT7 inhibition in the presence of increasing concentrations of OGT inhibitors, ST078925 and ST045849, and FUT 7 inhibitor, Gallic acid, respectively [26,54]. As shown in Figure 8, both GTs were inhibited by their corresponding inhibitors in a dose-dependent fashion, with an IC50 of 55, 58, and 0.6 µM for OGT’s ST078925, ST045849, and FUT7′s Gallic acid, respectively. These values were in a similar range to what was previously reported [26,54]. When 5 µM UDP or 10 µM GDP were incubated with serially diluted inhibitors and detected with UDP-Glo or GDP-Glo, respectively, there was no effect of the inhibitors on the nucleotide detection, suggesting that the bioluminescent assay reagents are not susceptible to interference by these chemicals. These bioluminescent detection assays were also shown to be robust, as they have been tested using 1280 chemicals present in the LOPAC compound library (data not shown). The robustness of the bioluminescent nucleotide detection assays demonstrated here for inhibitor studies is not surprising, as they contain similar core components as other bioluminescent assays previously developed for other enzymes, such as kinases, demethylases, and methyltransferases, and were successfully tested for chemical interference [37,49,55]. The combination between the use of a luciferase variant called Ultra-Glo and special reagent formulations proved to be essential for the resistance to chemical interference [37]. Together, these results indicate that the bioluminescent nucleotide assays for GT activity detection are robust with minimal compound interference, and therefore, they are suitable for inhibitor studies and high-throughput screening applications.

In summary, this report shows the development and characterization of homogeneous bioluminescent nucleotide detection methods that detect four nucleotides, UDP, GDP, UMP, and CMP, and demonstrated their utility in measuring nucleotide-sugar dependent glycosyltransferase activities. These assays are performed in a one-step “add and read” format, converting the nucleotide product of the GT enzymes into ATP, which is subsequently detected by a luciferase system to generate a bioluminescent signal. The UDP, GDP, and UMP/CMP detection methods detect the nucleotide from nanomolar concentrations to 25–50 µM. By detecting the activity of multiple GTs from several subfamilies, we demonstrated that nucleotide detection can be used as a universal method regardless of the acceptor substrate’s chemical nature. We also demonstrated that it could be used to determine substrate requirements, such as specificity and selectivity, for putative and known GTs, as well as to determine the apparent kinetic values of each of the donor and acceptor substrates used in the glycosyltransferase reactions. In addition, we showed the value of nucleotide detection in measuring the activity of GT enzymes with intrinsic nucleotide-sugar hydrolysis activity without an acceptor substrate and optimization of assay conditions, enabling the distinction between acceptor-dependent and -independent enzyme activity. Finally, our work demonstrates the usefulness of monitoring nucleotide formation as a method for studying glycosyltransferase inhibitors, and potentially identifying new GT compound inhibitors, and determining their mode of action and potency.

We believe that the universality and broad utility and ease of use of these nucleotide assays will enable the subsequent studies of members of the glycosyltransferase superfamily and may have a significant impact on diverse areas of Glycobiology research.

## 3. Materials and Methods

### 3.1. Glycosyltransferases and Substrates

Human UGT1A1 Supersomes™ were purchased from BD Biosciences (Woburn, MA, USA). The phosphoglycosyltransferase XcbA was generously provided by Dr. Willie Vann (FDA). The sOGT enzyme was produced in-house as follows. The sOGT sequence 2–236 was cloned into a vector with C terminal 6xHis, transformed into KRX cells, and the expression of the protein was induced with 0.1% Rhamnose at 23 °C overnight. The protein was purified via the HisLink™ Protein Purification System (Promega). All other enzymes were purchased from R&D Systems (Minneapolis, MN, USA).

Ultra-pure solutions of sugar donors UDP-Glc, UDP-Gal, UDP-GlcNAc, UDP-GalNAc, UDP-Glucuronic acid (GA), and GDP-Fucose were obtained from Promega Corporation (Madison, WI, USA). Powdered UDP-GlcNAc, UDP-GalNAc, and CMP-NeuAc were purchased from Sigma-Aldrich (Saint Louis, MO, USA), and powdered GDP-Fucose was purchased from Carbosynth (Berkshire, UK).

The acceptor substrates, biantennary N-linked core pentasaccharide, β1-3 Galactosyl-N acetylgalactosamine, and N-acetyl-D-lactosamine (LacNac), were purchased from V-Labs INC. (Covington, LA, USA). Glucose, Methyl-α-D-mannopyranoside, Fetuin, α-lactose Monohydrate, and estradiol were purchased from Sigma-Aldrich. N-Acetyl-D- glucosamine (GlcNAc) was obtained from EMD Chemicals Inc. (San Diego, CA, USA). Mucin 10 (153–165) EA2 Peptide and OGT peptide substrates were purchased from AnaSpec (Fremont, CA, USA). Recombinant human RhoA protein was from Creative BioMart (Shirley, NY, USA). NMX substrate was generously provided by Dr. Willie Vann (FDA).

### 3.2. Chemicals and Assay Components

The OGT inhibitors ST078925 and ST045849 were purchased from TimTec through Fisher Scientific. Alamithicin and Gallic Acid were from Sigma-Aldrich. White 96-well full and half volume assay plates (Catalog #s 3912 and 3693, respectively) were obtained from Corning Inc. (Kennebunk, ME, USA).

The UDP-Glo™, GDP-Glo™, and UMP/CMP-Glo™ glycosyltransferase assay kits from Promega Corporation are composed of a converting enzyme solution, a nucleotide detection reagent (made by mixing nucleotide detection buffer with an ATP Detection Substrate), and the corresponding nucleotide standards UDP, GDP, and UMP or CMP, respectively.

### 3.3. Bioluminescent Nucleotide Detection Protocol

The glycosyltransferase assay kits contain a specific enzyme solution that converts the corresponding nucleotide to ATP, which is used to generate a light signal. Briefly, in the UDP-Glo assay example, a UDP detection reagent is prepared by mixing the UDP-Glo enzyme solution with the nucleotide detection reagent. The UDP detection reagent is then added to the sample containing UDP in a 1:1 volume ratio, mixed, and then incubated for 1 h at room temperature (~23 °C). The UDP Detection Reagent converts in one enzyme-coupled step the UDP to ATP, then to light output using the luciferase/luciferin reaction. This luminescent signal is proportional to the amount of UDP present in the sample. The GDP-Glo and UMP/CMP-Glo assays are performed in a similar manner, using the same nucleotide detection reagent mixed with either GDP-Glo enzyme or UMP/CMP-Glo enzyme, respectively.

### 3.4. Nucleotide Standard Curves

Nucleotide standard curves were used to determine the sensitivity and linear range of the bioluminescent detection. UDP, GDP, UMP, or CMP standards were prepared in a generic glycosyltransferase (GT) buffer consisting of 50 mM Tris pH 7.5, 10 mM MgCl2, and 1 mM DTT. For the UDP standard curve example, a solution containing 25 µM of UDP was two-fold serially diluted in 12 wells of a 96-well plate to produce a dilution series from 25 µM to 0.024 µM plus a 0 µM UDP blank sample. Twenty-five microliters of each dilution were transferred to an assay plate, and UDP was detected using the UDP-Glo™ Assay following the manufacturer’s procedure. Briefly, 25 µL of UDP Detection Reagent was added to the standard curve samples and incubated for 60 min at room temperature (~23 °C) before the luminescence was recorded on a plate-reading luminometer. Similar conditions were used when GDP standard curves were generated, whereas, for UMP and CMP, the dilution series were performed from 50 µM to 0.048 µM.

### 3.5. Comparing Nucleotide-Sugars Purity

To test the effect of unpurified vs. ultra-purified sugar donors on the sensitivity of the bioluminescent assays, 100 µM purified or unpurified UDP-GlcNAc or UDP-GalNAc were mixed in separate solution with or without 300 nM UDP standard into GT buffer. As controls, 0 or 300 nM UDP solutions were prepared in GT buffer with no sugar substrates. Twenty-five microliters of the prepared solutions were transferred to separate wells of a 96-well plate and subjected to UDP detection. The luminescence was recorded, and the signal/background ratios were calculated by dividing the RLU values obtained from the samples containing UDP by the RLU values obtained from the corresponding 0 µM UDP sample. A similar experiment was performed on a UDP or GDP standard curve with 100 µM purified or unpurified UDP-GalNAc or GDP-Fucose, respectively. After UDP or GDP detection, the luminescence was recorded, and the signal/background ratios of the whole curves were plotted.

### 3.6. Glycosyltransferase Assay Conditions

Generally, all glycosylation reactions were carried out in 96-well white plates at 25 µL volumes using the buffers and substrates described in Table 2. For the enzyme titrations, glycosyltransferases were serially diluted in the corresponding buffer without substrates, and 12.5 µL were transferred to an assay plate. The reactions were started by the addition of 12.5 µL of a buffer solution containing either a 2x concentration of the corresponding donor and acceptor substrates or a 2x concentration of the corresponding donor substrate only. The substrates used, as well as reaction incubation time and temperature for each enzyme, are as described in Table 2 and each figure. Nucleotide formation was detected using the corresponding luminescent assay following the manufacturer’s procedure.

### 3.7. Donor and Acceptor Substrate Specificity Studies

For determining the preferences of glycosyltransferases for specific nucleotide-sugar donor substrates, 25 μL reactions were carried out in the corresponding GT buffer in the presence of 83 µM of each of the UDP-sugars -Gal, -Glc, -GlcNAc and -GalNAc, and 0.25 ng of β4GalT1 with 10 mM GlcNac as a substrate acceptor, 18 ng β4GalT2 with 10 mM Glucose, 2 ng GALNT1 with 0.5 mM Mucin EA2 peptide, 100 ng GALNT4 with 0.5 mM Mucin EA2 peptide, and 2.5 ng OGT with 50 µM OGT-peptide substrate. For titrating the UDP-sugars in a β4GalT1 reaction, 25 μL reactions were carried out containing 15 ng of β4GalT1 with 10 mM GlcNac and a dilution series from 0.5 to 0.008 mM for each of the UDP-sugars. For determining the preferences of a glycosyltransferase for a specific acceptor substrate, 25 μL reactions were carried out as titration of the substrates in an MGAT-III reaction containing 30 ng of MGAT-III with 1 mM UDP-GlcNAc and a dilution series from 2 to 0.03 mM of different sugar-acceptor substrates of different chemical structure. The reactions were incubated for 1 h at 23 °C. UDP formation was detected using a UDP-Glo assay following the manufacturer’s procedure.

### 3.8. Substrate Km Determinations

For determining the glycosyltransferases, Km for sugar donor and acceptor substrates, 25 µL reactions were performed with the amount of enzyme and substrates described in the figures for each GT. After the indicated incubation times, 25 μL of the corresponding detection reagent was added to the reactions and incubated for 60 min at 23 °C before the luminescence was recorded. A standard curve for each nucleotide was performed at the same time to calculate the amount of nucleotide produced per minute per microgram protein. The Km values were extracted from the data after fitting to the Michaelis-Menten equation using the non-linear regression fit in GraphPad Prism^®^, version 9.

### 3.9. Detection of Acceptor Substrate Dependent and Independent Enzyme Activity

In order to assess the levels of nucleotide sugar hydrolysis in the presence and absence of acceptor substrate, FUT2 and FUT7 were titrated in 25 µL reactions in the presence of 40 µM Ultra-pure GDP-Fucose. The FUT2 reactions were performed in FUT2 buffer at 37 °C for 30 min in the presence of 0, 0.04, 2, or 10 mM of the acceptor substrate α-Lactose. The FUT7 reactions were performed in FUT7 buffer at 37 °C for 30 min in the presence of 0, 1, 2, or 20 µM of the acceptor substrate Fetuin. After the indicated incubation times, GDP formation was detected using a GDP-Glo assay following the manufacturer’s procedure.

### 3.10. OGT and FUT7 Inhibition with Chemical Compounds

To determine the use of bioluminescent nucleotide detection assays for GT inhibitor studies, OGT and FUT7 activities were assessed in the presence of dose responses of known inhibitor compounds, ST078925 and ST045849 for OGT and Gallic acid for FUT7. OGT compounds were dissolved in DMSO at a stock concentration of 25 mM, and Gallic acid was prepared in water at a stock concentration of 10 mM. For OGT inhibition, the compounds were serially diluted three-fold from 500 to 0.025 μM in a 25 µL reaction containing 0.1 ng/μL OGT, 50 μM OGT-peptide substrate, and 100 µM UDP-GlcNAc in OGT buffer with a 1% final concentration of DMSO. A second set of reactions containing 5 µM UDP instead of OGT were used as a control for assessing the inhibition of the assay reagents by the compounds. For FUT7 inhibition, Gallic acid was serially diluted two-fold from 125 to 0.015 μM in a 25 µL reaction containing 0.6 ng/μL FUT7, 10 μM Fetuin, and 40 µM GDP-Fucose in FUT7 buffer. A second set of reactions containing 10 µM GDP instead of FUT7 was used as a control for assessing the inhibition of the assay reagents by the Gallic acid. The OGT and FUT7 reactions were incubated for 60 min at 23 °C and 30 min at 37 °C, respectively. After the indicated incubation times, UDP and GDP formation was detected using the corresponding Glo assay following the manufacturer’s procedure.

### 3.11. Signal Detection and Data Analysis

All 96-well assay plates were read using a GloMax^®^ 96 Microplate Luminometer from Promega. The instrument was set to 0.5 s integration time. To plot, analyze the data, and calculate all enzyme reaction biochemical values, both Microsoft Excel and GraphPad Prism^®^, version 9 Software were used. IC_50_ values were determined by using a non-linear regression fit to a sigmoidal dose-response (variable slope).

## Figures and Tables

**Table 1:** Sensitivity (signal to background ratios) of the bioluminescent nucleotide assays.

UDP-Glo Assay	Signal to Background Ratios (Fold) at Each Nucleotide Concentration (µM) ^1^											
	25	12.5	6.25	3.13	1.56	0.78	0.39	0.20	0.10	0.05	0.02	0
**UDP**	12,368	6803	3588	1828	917	459	227	119	60	30	16	1
	*441.7*	*284.6*	*153.4*	*76.2*	*38.7*	*17.1*	*10.6*	*5.3*	*3.0*	*1.0*	*0.6*	*0*
**CDP**	12,378	7086	3921	2012	1040	507	255	124	61	31	16	1
	*44.6*	*51.5*	*103.0*	*50.2*	*32.0*	*22.3*	*8.7*	*5.8*	*2.0*	*1.6*	*0.5*	*0*
**GDP-Glo assay**	**Signal to background ratios (fold) at each nucleotide concentration (µM) ^1^**											
	**25**	**12.5**	**6.25**	**3.13**	**1.56**	**0.78**	**0.39**	**0.20**	**0.10**	**0.05**	**0.02**	**0**
**GDP**	41,700	24,917	13,317	7028	3533	1788	898	436	208	110	54	1
	*2139.8*	*1848.1*	*338.0*	*446.7*	*53.0*	*77.6*	*11.6*	*32.0*	*15.5*	*7.2*	*4.2*	*0*
**UMP/CMP-Glo assay**	**Signal to background ratios (fold) at each nucleotide concentration (µM) ^1^**											
	**50**	**25**	**12.5**	**6.25**	**3.13**	**1.56**	**0.78**	**0.39**	**0.20**	**0.10**	**0.05**	**0**
**UMP**	1922	1009	535	259	139	68	34	18	9	5	3	1
	*32.33*	*1.70*	*2.53*	*4.40*	*1.40*	*0.53*	*0.48*	*0.21*	*0.09*	*0.07*	*0.05*	*0*
**CMP**	2186	1128	595	308	166	83	40	21	11	6	3	1
	*41.40*	*22.03*	*9.75*	*10.62*	*2.74*	*2.35*	*1.17*	*0.87*	*0.40*	*0.06*	*0.16*	*0*

^1^ Signal to background ratios represents the mean of three replicates. The numbers below SBs represent the standard error values for each SB point derived from the titration.

**Table 2:** Buffers and substrates used for the Glycosyltransferase reactions.

Glycosyltransferase	Buffer	Donor	Acceptor	Temp.	Time (min)
**MGATIII** N-Acetylglucosaminyl-transferase III	50 mM Hepes 6.8, 5 mM MnCl2	UDP-GlcNAc	Biantennary-N-linked core pentasaccharide	23 °C	60
**β4GalT1** β-1, 4-Galactosyl-transferase 1	50 mM Tris 7.5, 5 mM MnCl2, 1 mM DTT	UDP-Gal	GlcNAc	23 °C	60
**β4GalT2** β-1, 4-Galactosyl-transferase 1	50 mM Tris 7.5, 5 mM MnCl2, 2 mM CaCl2	UDP-Gal	Glucose	23 °C	60
**GALNT1** Polypeptide GalNAc Transferase 1	50 mM Tris 8.0, 2.5 mM MnCl2, 1 mM CaCl2, 1 mM DTT	UDP-GalNAc	Mucin EA2 peptide	37 °C	60
**GALNT4** Polypeptide GalNAc Transferase 4	25 mM Tris 7.5, 5 mM MnCl2, 2.5 mM CaCl2	UDP-GalNAc	Mucin EA2 peptide	37 °C	60
**TcdB** C. difficile Toxin B Protein	50 mM Hepes 7.5, 100 uM KCl, 2 mM MgCl2, 2 mM MnCl2, 1 mM DTT	UDP-Glc	RhoA protein	23 °C	60
**OGT** O-GlcNAc Transferase	25 mM Tris 7.5, 12.5 mM MgCl2, 0.062 mg/mL BSA, 1 mM DTT	UDP-GlcNAc	OGT-peptide substrate	23 °C	60
**UGT1A1** Glucuronosyltransferase 1A1	50 mM TES, 8 mM MgCl2, 25 mg/mL Alamethicin, 15 mM NaF pH 7.5	UDP-GA	Estradiol	37 °C	60
**FUT2** Fucosyltransferase 2	5 mM Tris 7.5, 30 mM NaCl2, 2 mM MnCl2, 2 mM CaCl2	GDP-Fucose	α-lactose	37 °C	30
**FUT3** Fucosyltransferase 3	5 mM Tris 7.5, 1 mM MnCl2	GDP-Fucose	LAcNAc	23 °C	60
**FUT7** Fucosyltransferase 7	20 mM Tris 7.5, 2 mM MnCl2, 2 mM CaCl2	GDP-Fucose	Fetuin	37 °C	30
**Xcb A** Meningococcal X capsule N-acetylglucosamine-1-phosphotransferase	50 mM Hepes 7.5, 25 mM MgCl2, 100 mM NaCl2, 2.4 mM imidazole	UDP-GlcNAc	NMX (α1→4)-linked GlcNAc-1-phosphate polymer	23 °C	60
**ST6Gal1** β-galactoside α-2,6-sialyltransferase 1	5 mM Tris 7.5, 150 mM NaCl2, 5 mM CaCl2, 5 mM MnCl2	CMP-NANA	LAcNAc	23 °C	60

## Data Availability

Not applicable.
